# Correlation of the SLAP lesion with lesions of the medial sheath of the biceps tendon and intra-articular subscapularis tendon

**DOI:** 10.4103/0019-5413.55974

**Published:** 2009

**Authors:** William F Bennett

**Affiliations:** Bennett Orthopedics & Sports Medicine, 1250 S. Tamiami Tr., Suite 303, Sarasota, FL. 34242, USA

**Keywords:** Instability, SLAP lesion, medial sheath lesion, biceps tendon, intra-articular subscapularis tendon

## Abstract

**Background:**

Superior labral anterior to posterior (SLAP) lesions have been well described in the literature and are thought to be secondary to traction injuries to the biceps anchor and/or falls on the outstretched arm. The pulley has recently been described as a structure that aids in the prevention of biceps instability. The intra-articular subscapularis insertion (IASS) has been noted to contribute to the robust nature of the medial sheath. The purpose of the study was to determine a potential correlation of SLAP lesions and pulley lesions with/without IASS lesions, (hereafter referred to as medial sheath) as forces that can disrupt the biceps anchor and may also disrupt structures of the medial sheath or vice-versa.

**Materials and Methods:**

Three hundred and sixteen consecutive shoulder arthroscopies performed by one surgeon were reviewed retrospectively. Operative reports and arthroscopic pictures were carefully reviewed with particular attention paid to the labral and pulley pathology. Selection bias was noted as the author had never operated primarily for a Type 1 SLAP lesion. Following, however, and as such, the exclusion criteria, was a Type 1 SLAP.

**Results:**

There were a total of 30 SLAP lesions and a total of 126 medial sheath lesions. There were 13 patients who had both SLAP and medial sheath lesions. There were 17 patients who had a SLAP lesion without a medial sheath lesion. There were 96 medial sheath lesions without a SLAP. A comparison of rates between patients who had a medial sheath lesion with a SLAP and those who had a medial sheath lesion without a SLAP, for the 316 patients, and when tested with a Fisher exact test revealed that there was no statistical significance, *P* = 0.673. The prevalence of SLAP lesions in this population of 316 patients was 9.4%, Buford 1%, medial sheath lesions 39%, and SLAP and medial sheath lesions 4%. Interestingly, there were three Buford complexes, all associated with a SLAP and one Buford complex was associated with both a SLAP and a pulley.

When looking at the rate for medial sheath lesions when restricted to patients with SLAP lesions, the medial sheath lesion rate was 43.3% (13/30; 95% confidence interval 19.6–66.9%). The medial sheath lesion rate for patients with SLAP lesions differs from a rate of zero and is statistically significant, with a *P* value <0.05. In other words, when a SLAP lesion is present there is a statistically significant rate of medial sheath lesions, a previously unpublished association.

**Conclusions:**

With a 43% association of the medial sheath lesion with SLAP lesions, the author postulates that forces that affect the biceps anchor may also damage the pulley system of the bicipital sheath and, as such, this anatomic structure should be evaluated, especially when SLAP lesions are present.

## INTRODUCTION

Injury to the superior labrum i.e. superior labral anterior to posterior lesion (here after referred to as a SLAP), has been described to occur secondary to traction,^1^ compression with the shoulder flexed and abducted,^2^ possibly with a “peel-back” phenomenon occurring during the late cocking phase of the throwing motion^3^. Some SLAP lesions, especially those found in patients over the age of 40, may represent an evolving acquired event, as DePalma has suggested for rotator cuff tears.^4^ Clearly, the SLAP lesion can be created from a variety of factors and/or forces.

The pulley lesion has been described^5^–^9^ with its variations. The lesion most often consists of injury to the medial wall of the bicipital sheath, although the lesion can affect the lateral bicipital sheath as well. The above authors have noted the association of the medial sheath lesion with various injuries to the rotator cuff tendons and, in particular, it occasionally is found with a lesion of the intra-articular subscapularis tendon (IASS). The pulley proper is the reflection pulley consisting of the confluence of the superior glenohumeral ligament (SGHL) with the medial coracohumeral ligament (mCHL). While the superior insertion of the subscapularis, IASS, is not a part of the pulley “proper,” it contributes significantly to the medial support of the biceps tendon and contributes to the medial sheath of the bicipital groove.

There has been an association of the Buford complex with SLAP lesions.^10^ To date, there has been no published study that has looked at the association of the SLAP lesion with the medial sheath lesion. Certainly, forces that can disrupt the biceps anchor may affect the medial wall of the bicipital sheath.

The purpose of this study was to determine if there was any correlation between the findings of a SLAP lesion with lesions of the medial sheath of the biceps tendon, pulley with/without IASS.

## MATERIALS AND METHODS

A retrospective review of 316 consecutive arthroscopies, performed by one surgeon, were subjected to analysis. Operative reports, arthroscopic pictures and a video review were undertaken. However, majority of the findings were entered into a database concurrent with the actual surgical procedure. Careful attention was paid to the labral pathology and structures surrounding the bicipital sheath. Selection bias was noted as no SLAP Type 1 lesions were included in this study. Subtypes of SLAP lesions were categorized according to the work of Snyder.^11^ To qualify as a SLAP lesion, i.e., more than just some labral fraying, the biceps anchor had to be pulled away from the superior glenoid by more than 5 mm, the biceps anchor had to be unstable to neuroprobe palpation, and there had to be the appearance of granulation tissue, significant fissuring, and fraying to be classified as a Type 2 lesion.

All patients had pre-operative magnetic resonance (MR) arthrograms, which indicated the SLAP lesion. The MR arthrogram was not as helpful in elucidating whether there was a lesion of the pulley with/without IASS lesions. An intra-operative evaluation of the biceps anchor and the area of the medial sheath was undertaken with an arthroscope and neuroprobe. The biciptal sheath was evaluated and lesions of the IASS, reflection pulley (medial sheath), and lateral sheath were recorded and classified according to the work of Habermeyer^7^ and Bennett.^5^,^12^

The mean age of the patients was 34, the range being 18–48 years. There were four categories of patients noted in this series; overhead athletes (n=6), trauma/falls (n=14), automobile accidents (n=6), and no history of injury (n=4).

All patients presented with shoulder pain. Physical exam included observation, palpation, strength testing, and specific tests for biceps inflammation/instability, instability, and SLAP lesions. Biceps inflammation was most often noted by direct palpation of the biceps in the bicipital groove and not the Speeds test. Biceps subluxation signs and Yergason's and the passive subluxation tests were variable. The SLAP signs and Obrien's and load and shift tests were uniformly positive. However, the Obrien test was often noted to be positive in patients who had lesions of the medial sheath without a SLAP lesion.

While the operative reports, pictures, and videos were reviewed to gather detailed information of the subjects, the operative findings were entered contemporaneously over time into the practice's database and, as such, the review was performed mainly for distinguishing the structures involving the medial sheath.

Statistical analysis was performed using a Fisher exact test. Confidence intervals were developed for all patients who had a SLAP and a medial sheath lesion with/without IASS involvement and for all patients who had a medial sheath lesion with/without IASS involvement that did not have a SLAP.

## RESULTS

There were a total of 30 SLAP lesions and a total of 126 lesions of the medial sheath to include pulley with/without IASS lesions and IASS lesions alone. There were 13 patients who had both a SLAP and a medial sheath lesion with/without IASS or IASS alone. There were 17 patients who had a SLAP lesion without a medial sheath lesion with/without IASS or IASS alone. There were 96 medial sheath lesions with/without IASS or IASS alone without a SLAP.

There were 28 Type 2 SLAP lesions and two Type 3 SLAP lesions. Type 1 SLAP lesions were excluded as simple fraying and degeneration of the labrum was not felt to be significant enough to classify as an injury thus introducing some selection bias.

A comparison of rates between patients who had a medial sheath lesion with a SLAP and those who had a medial sheath lesion without a SLAP, for the 316 patients, and when tested with a Fisher exact test revealed that there was no statistical significance, *P* = 0.673.

The prevalence of SLAP lesions in this population of 316 patients was 9.4%, Buford lesion 1%, medial sheath lesions with/without IASS or IASS alone 39%, and SLAP with medial sheath lesions with/without IASS or IASS alone 4%. Interestingly, there were three Buford complexes, all associated with a SLAP and one Buford complex associated with both a SLAP and a pulley.

When looking at the rate for medial sheath lesions with/without IASS or IASS alone when restricted to patients with SLAP lesions, the medial sheath lesion rate with/without IASS or IASS alone was 43.3% (13/30; 95% confidence interval 19.6–66.9%). The medial sheath lesion with/without IASS or IASS alone rate for patients with SLAP lesions differs from a rate of zero and is statistically significant, with a *P* 0 value <0.05. In other words, when a SLAP lesion is present, there is a statistically significant rate of medial sheath lesions with/without IASS or IASS alone (henceforth medial sheath lesion), a previously unpublished association.

**Figure 1:**
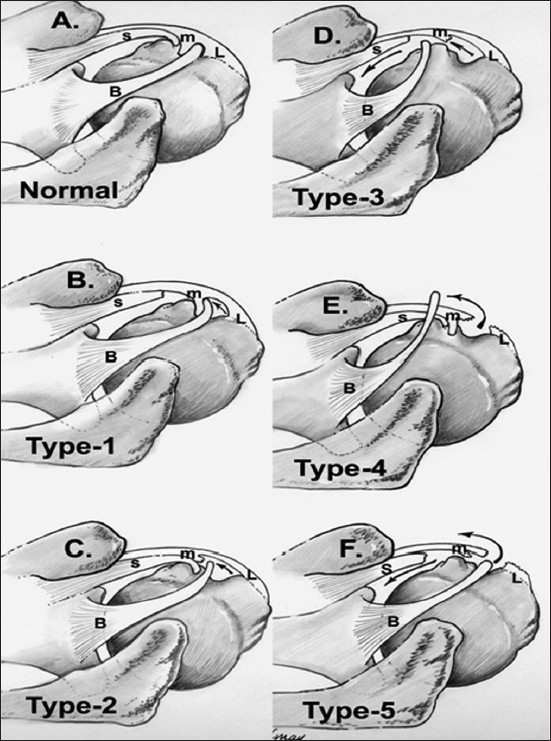
Various lesions of the bicipital sheath: Type 1- Intra-articular subscapularis (IASS) lesion alone, Type 2- Medial pulley alone, Type 3- Both IASS and medial pulley, Type 4- Lateral pulley alone, Type 5- Lesion of both medial and lateral pulley with IASS

Those patients who had both a SLAP and a medial sheath lesion had lesions of the medial sheath. There were no lesions of the lateral sheath. Of the medial sheath lesions, four had IASS tears - without injury to the medial pulley (Type 1 biceps instability), four had medial sheath lesions only (Type 2 biceps instability) - and five had lesions of both (Type 3 biceps instability)^5^,^6^,^12^ [Figure 1].

## DISCUSSION

Various postulates have been proposed to explain the mechanism of injury to the biceps anchor. It is clear that various mechanisms and forces contribute to this injury. The results of this study confirm that there is an association of medial sheath lesions with/without IASS or IASS alone with the SLAP lesion that is statistically significantly different from zero. This finding suggests that there are forces during an injury that can affect both the biceps anchor and the pulley.

It has been shown by various authors^5^–^9^ that the degenerative process of rotator cuff disease may include the medial sheath, to include the IASS, reflection pulley, and, in rarer circumstances, the lateral sheath. As such, medial sheath lesions are found in degenerative and instability conditions.

These lesions have been classified based on arthroscopic findings^6^–^9^,^12^ and various mechanisms for injury to the medial sheath based on their respective findings have been proposed. When the arm is in the abducted and externally rotated position, the biceps is forced against the medial sheath and in internal rotation, the lateral sheath.

This arm position is the same as the position for various postulated injury mechanisms,^1^–^3^ dynamic arthroscopic assessment of the peel back mechanism^3^ (ABER position), dynamic biceps instability,^9^ and, the greatest, “load to failure.”^13^ Seemingly, with this study's noted association, it may be reasonable to conclude that this position may also place the medial pulley at risk with various forces.

Three types of internal impingement have been postulated in the shoulder; anterior impingement,^14^ anterosuperior impingement (ASI), and posterosuperior impingment (PSI).^3^ With ASI, the arm is adducted and the shoulder is in internal rotation. It has been shown that the pulley abuts the anterior glenoid, possibly contributing to injury of the pulley in extremes of motion. With the shoulder in abduction and external rotation, the peel back phenomenom has been shown to accentuate the posterior portion of a SLAP.^3^ and PSI, and some have observed the pulley to abut the posterosuperior glenoid.^15^

Physiologically, the long head of the biceps is thought to be an anterior stabilizer of the glenohumeral joint during rotation of the shoulder.^2^ The biceps is known to be an active dynamic stabilizer in the throwing athlete.^1^ Overhead athletes fare less well than non-throwers with the ability to return to pre-injury level of activity following repair of a SLAP lesion.^16^ Other authors have noted the less-than-desirable return to pre-injury activity in this population.^17^

While both ASI and PSI are postulated as entities that are responsible for shoulder internal derangement and dysfunction, it is equally likely that biceps instability occurs with progressive injury to the pulley thus creating a symptomatic tenosynovitis, fraying with progressive disease of the long head of the biceps tendon.

The associations of bicipital tenosynovitis, tendonitis, fraying, subluxation, and instability have been noted with lesions of the medial wall of the bicipital sheath.^5^,^6^–^9^,^12^ These lesions, if not repaired, may allow for the biceps tendon to continue to be irritated or frayed on the supratubercular ridge, allowing for continued symptoms. This study has shown that when a SLAP lesion is present, there is a statistically significant association of having a concomitant medial sheath lesion.

Habermeyer^7^ noted that the superior glenohumeral ligament (SGHL) is the most important structure for stabilizing the long head of biceps (LHB). Itoi^18^ noted that the LHB stabilized the humeral head to anterior translation with the arm in abduction, with the arm externally rotated at 60° and 90°. Finally, Werner^19^ showed that lesions of the pulley lead to anterior instability of the long head of the biceps in external rotation. Finally, other studies have shown a high incidence of injury to the LHB with medial sheath lesions.^5^,^12^ This author postulates that this noted association and the simple repair of the SLAP lesion, or non-repair of the pulley pathology when a medial sheath lesion is present, may account for the noted less-than-favorable outcome in various patient populations who have had a SLAP repair. Various studies and their conclusions lend credence to this postulate.

While this study has shown this association, the hypothesis proposed for the “less-than-desirable outcomes” in certain patient populations is strictly speculative. Certainly, continued tightness of the posterior capsule, continued anterior instability, failure of repair, and loose fixation devices are alternative explanations. However, this association introduces another hypothesis for the origin of symptoms surrounding lesions of the biceps tendon, i.e., is it insertional pain at the biceps anchor site or pain from disruption of the pulley with concomitant bicipital tenosynovitis and/or fraying and subluxation/instability at the supratubercular ridge, or is it both that contribute to disability in certain patient populations.

## CONCLUSION

The findings of this study show that when a SLAP lesion is present, there is a statistically significant finding of medial sheath lesions. With a 43% association of the medial sheath lesion with SLAP lesions, the author postulates that forces that affect the biceps anchor may also damage the pulley system of the bicipital sheath and, as such, this anatomic structure should be evaluated, especially when SLAP lesions are present and repaired.
